# Application of visual transformer in renal image analysis

**DOI:** 10.1186/s12938-024-01209-z

**Published:** 2024-03-05

**Authors:** Yuwei Yin, Zhixian Tang, Huachun Weng

**Affiliations:** 1 The College of Health Sciences and Engineering, University of Shanghai for Science and Technology, 516 Jungong Highway, Yangpu Area, Shanghai, 200093 China; 2 The College of Medical Technology, Shanghai University of Medicine & Health Sciences, 279 Zhouzhu Highway, Pudong New Area, Shanghai, 201318 China

**Keywords:** Deep learning, Transformer, Convolutional neural network, Attention mechanism, Kidney disease

## Abstract

Deep Self-Attention Network (Transformer) is an encoder–decoder architectural model that excels in establishing long-distance dependencies and is first applied in natural language processing. Due to its complementary nature with the inductive bias of convolutional neural network (CNN), Transformer has been gradually applied to medical image processing, including kidney image processing. It has become a hot research topic in recent years. To further explore new ideas and directions in the field of renal image processing, this paper outlines the characteristics of the Transformer network model and summarizes the application of the Transformer-based model in renal image segmentation, classification, detection, electronic medical records, and decision-making systems, and compared with CNN-based renal image processing algorithm, analyzing the advantages and disadvantages of this technique in renal image processing. In addition, this paper gives an outlook on the development trend of Transformer in renal image processing, which provides a valuable reference for a lot of renal image analysis.

## Background

Kidney disease is a series of infections caused by kidney damage in function, morphology, or structure. Common kidney diseases include glomerulonephritis, pyelonephritis, diabetic nephropathy, hypertensive nephropathy, kidney stones, etc. Glomerulonephritis and diabetic nephropathy are the leading causes of chronic kidney failure. Today, ten percent of the world's population suffers from chronic kidney disease (CKD), which has become one of the most prevalent and fatal diseases and seriously affects people's health [1]. Kidney stones disease (KSD) is a common disease caused by solid mineral deposits that form in the kidneys [2]. According to the World Health Organization, approximately 5–10% of the global adult population suffers from kidney stones, with 10% and 14% in some developed countries in Europe and North America, respectively [3]. Meanwhile, kidney stones have been on the rise in the past decades. Renal cancer is a common urological malignancy, with more than 4 million new cases diagnosed yearly [4]. Therefore, improving the accuracy of diagnosis and early detection rate of nephrolithiasis is very important for the treatment and prognosis of patients.

With the development of digital medical technology, medical image processing technology has also been rapidly developed and has become one of the crucial tools in the medical field, especially in diagnosing renal diseases. Several medical imaging modalities exist, such as ultrasonography, computed tomography (CT) [5], and magnetic resonance imaging (MRI) [6]. However, imaging tests may require longer scanning times, and diagnostic images need more time and effort from healthcare professionals. Long-term fatigue of healthcare workers is likely to result in subjective misdiagnosis or underdiagnosis.

Some studies have shown that using machine learning in medical imaging can reduce the possibility of diagnostic errors and thus effectively improve diagnostic accuracy [2]. Therefore, improving the ability and automation of image analysis is a widespread issue in medical research today. Deep learning, as a branch of machine learning, has been tried to be applied in diagnosing CKD and predicting the decline of renal function [7], renal insufficiency, and diabetic nephropathy.

Deep Self-Attention Network (Transformer), as a new type of sequence model, has been widely recognized for its excellent performance in fields such as natural language processing [8]. Kidney CT/MRI images are sequential structural data with complex structural correlations between different parts. The transformer can simultaneously learn the contextual information of other parts of kidney images through the mechanism of multi-attention and capture the global structural relationship of the images more comprehensively and accurately to improve the recognition effect. The focus of current research is how to introduce it into medical image processing, especially in kidney disease. Moreover, the Transformer framework is more general, and the trained base model can be used for other renal image analysis tasks, such as classification, detection, segmentation, etc. This paper outlines the current stage of the Transformer's application in kidney image classification, segmentation, and detection and compares it with traditional CNN models.

## Introduction to transformer

**Fig. 1:**
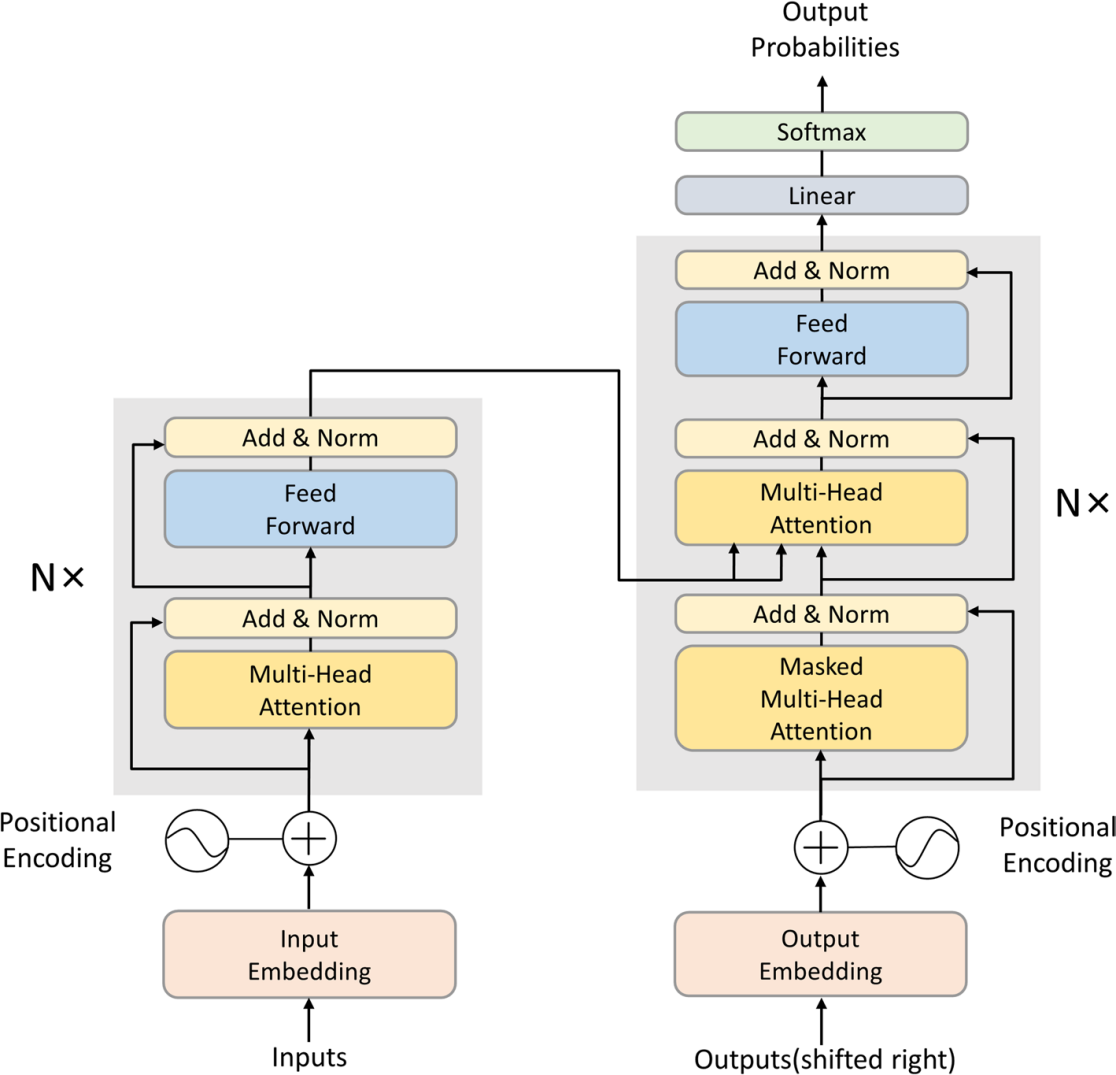
Basic structure of transformer

The Transformer model is the first transduction model that relies exclusively on self-attention to compute its input and output representations without recurrent neural networks (RNNs) or CNNs for sequence comparison [8]. Compared to commonly used models such as RNNs and CNNs, Transformer has a higher parallel computation capability due to an attentional mechanism that simultaneously allows the computation to consider all input words or characters. Moreover, the self-attention mechanism can effectively handle long sequential data and improve the modeling ability of long-range dependencies. The transformer abstracts the encoder and decoder into individual modules (as shown in Fig. 1). In the encoder, the inputs are mapped to a multidimensional space, and the input representation is learned through the multi-head self-attention mechanism. The feed-forward neural network uses the ReLU transform for the nonlinear transformation. In the decoder, the model also uses a standard attentional mechanism to compute the attentional weights between the input and its corresponding context for the decoding operation.

### Vision transformer

Vision transformer (ViT) is the application of Transformer models to computer vision, especially for image classification tasks. ViT transforms images into sequences by segmenting them into different paths and encodes and classifies them using standard Transformer models. Compared to traditional CNN models, ViT is based on a multi-head self-attention mechanism [9], which can adapt to inputs of different sizes and shapes, provides better flexibility, and allows migration learning after large-scale pre-training.

#### Self-attention

Self-attention is a unique mechanism for computing the interaction between two elements in a sequence. Given a sequence input, each element can be used simultaneously as a query, key, and value. The attention function can be described as mapping a query and a set of key-value pairs to an output, where the query, key, value, and production are vectors. The outcome is computed as a weighted sum of values, where the weights assigned to each value are calculated by the compatibility function of the query with the corresponding key [8]. These weights can be used in a weighted sum to add the encoded vector at that location.

#### Multi-head attention

Multi-head attention is a combination of multiple sets of self-attention mechanisms, each mapping between a pair of queries, keys, and values, thus allowing the model to simultaneously attend to different representation subspaces from other locations [8]. In this process, multiple attention mechanisms form a "head", each getting a separate set of queries, keys, and values and generating the corresponding output [10]. It captures multiple feature representations simultaneously and detects the relationship between different parts, thus developing more contextual relevance and significantly improving the model's ability to learn the original drawing.

### Other techniques

Recent studies have found that multilayer perceptual network (MLP) models excel in solving image tasks without convolution or self-attention mechanisms. Such models learn representations only through basic linear algebra operations, which can be computed repeatedly for different spatial locations and feature channels. Despite the long-term dominance of CNNs and ViT, simple MLP models perform well on specific kidney image processing tasks. This demonstrates that MLPs can learn efficient representations, opening up new ideas for deep learning. A typical example is that Saikia et al. [11] proposed a model MLP-UNet based only on MLP architecture for glomerular segmentation tasks. The results show that MLP-UNet performs on PAS-stained whole kidney images comparable to the pre-trained model TransUNet but with a 20% reduction in the number of parameters without needing pre-training. The research advancement of MLP models has proposed many novel architectures such as gMLP [12], ResMLP [13], ASMLP [14], Cyclemlp [15], etc. Transformer, CNN, and MLP perform differently on different tasks, and there is yet to be a unified optimal structure for deep learning. This section will focus on applying Transformer and its variant architectures to the kidney image processing task to find a network architecture more suitable for a specific task and thus advance the field.

## Application of transformer in renal image processing

Currently, the Transformer mechanism has more applications in renal image processing, mainly including image classification [16], tumor lesion segmentation [17], renal organ segmentation [18, 19], etc. In addition, Transformer can achieve prognostic assessment of renal diseases [20, 21], provide treatment plans [22], help doctors write pathology reports [23], construct electronic medical records [24], and so on.

Using the Transformer mechanism, the application that can be used in renal image processing can realize the fast and accurate automated analysis and processing of renal images, improve clinicians' efficiency and diagnosis level, and bring new opportunities and challenges for renal disease research and clinical treatment.

### Transformer applied to kidney image segmentation

Renal cancer is now considered one of the most common malignant tumors in urology, leading to a large number of deaths every year [19]. In the past 30 years, the number of new cases of renal cancer in China has dramatically increased from 110,700 to 598,300 cases [25]. Traditionally, the lesion areas of renal cancer patients are mainly identified by clinicians' depiction, which relies heavily on the clinical experience of doctors and is very time-consuming and prone to erroneous judgment. Accurate measurements from medical images can help doctors make accurate diagnoses and provide timely treatment. Medical image segmentation aims at identifying tumors and depicting different sub-regions of an organ from the corresponding background by assigning labels of predefined categories to each pixel in a medical image, e.g., CT [5] MRI [6]. Therefore, the emergence of automatic medical image segmentation techniques is crucial to improve the accuracy and efficiency of clinical diagnosis.

CNN-based and U-Net-based [26] medical image segmentation algorithms have performed better in recent years. Still, based on the limitations of convolutional operations, they cannot capture long-range relationships. To solve this problem, there have been some research works applying network models based on Transformer with improvements to kidney image segmentation and have achieved good results. In renal image segmentation, more application scenarios are renal organ segmentation, renal lesion segmentation, and automatic target area outlining.

In this paper, the goodness of segmentation performance is usually expressed in terms of the following metrics. Dice similarity coefficient (DSC): measures the overlap between the segmentation result and the ground truth. Hausdorff distance (HD): computes the maximum distance between two sets, assessing differences between the predicted boundary and the ground truth boundary. IOU (Intersection over Union): calculates the ratio of the intersection area to the union area of the predicted region and ground truth, reflecting the degree of overlap. MIoU (mean IOU): represents the average IOU values of multiple samples, offering a comprehensive evaluation of model performance. F1 Score: considers both precision and recall, providing a balanced assessment of classification model performance. AUC (area under the ROC curve): reflects the overall performance of a classification model by measuring the relationship between true and false positive rates at different thresholds. Accuracy (ACC): indicates the model's overall classification performance. Sensitivity: measures the correct identification rate of positive cases. Specificity: measures the correct identification rate of negative cases.

Models for renal image processing based on transformers typically employ simple random rotation data augmentation for preprocessing, using cross-entropy as the loss function and optimization methods such as SGD and Adam. Regularization techniques include dropout and weight decay. Key hyperparameters encompass the learning rate (usually ranging from 1e−4 to 1e−5), batch size (4 to 16), and dropout rate (0.1 to 0.3)[27].

#### Multi-organ segmentation of the abdomen

**Fig. 2:**
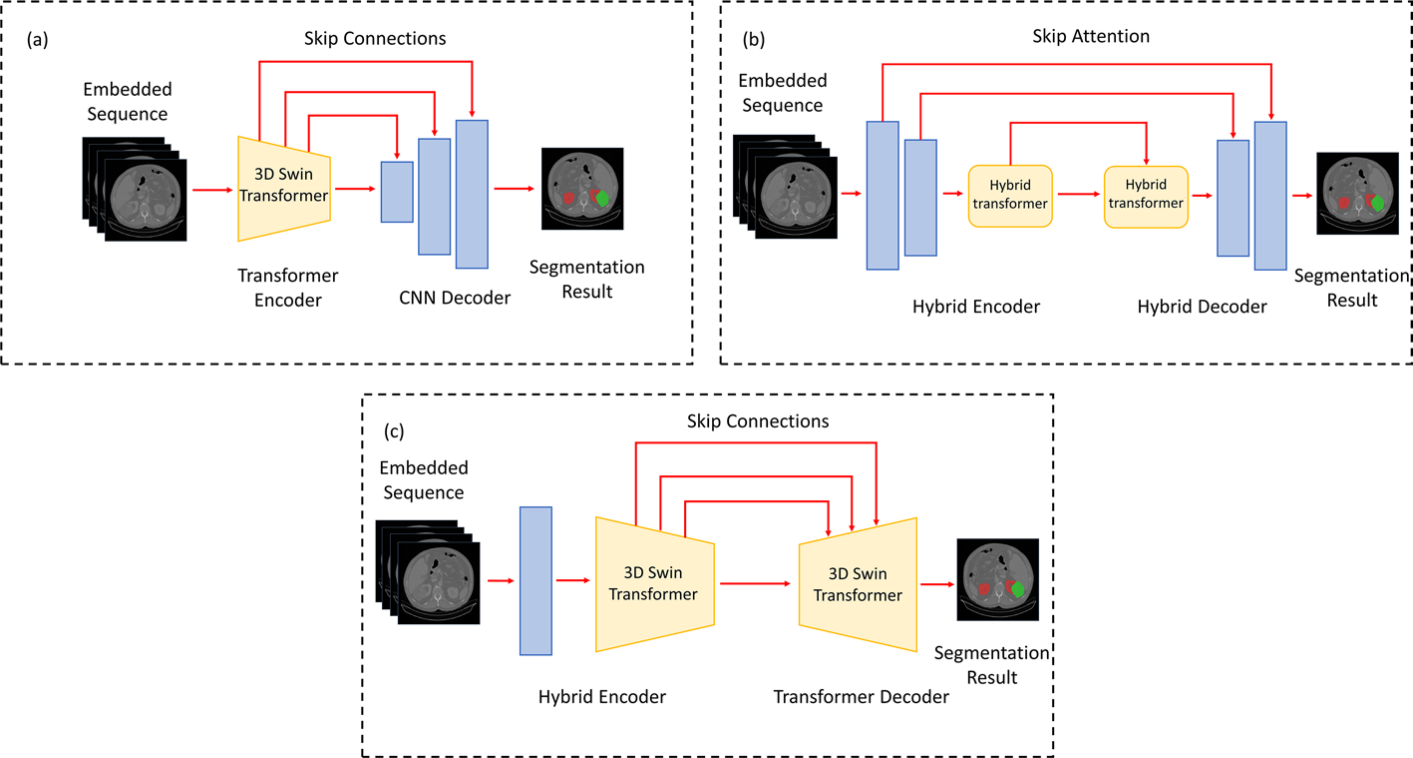
**a**–**c** Shows the transformer model-based encoder method, the method of using the transformer model between encoder and decoder, and the decoder based on the transformer model

Accurate kidney organ segmentation can provide clinicians with important information, and the task is often integrated with abdominal multi-organ segmentation. In the abdominal multi-organ segmentation task, the algorithm needs to segment all the organs in the abdomen at once. The synapse dataset (https: //doi.org/10.7303/syn3193805) is the most common publicly available dataset for abdominal multi-organ segmentation. Previous researchers usually use CNN for multi-organ segmentation [28]. The algorithm needs to consider global and local information to improve further the segmentation accuracy, which led to a combined Transformer–CNN model. According to the way of combining CNN and Transformer, hybrid Transformer model methods are usually classified into three categories (as shown in Fig. 2):

①Methods based on the encoder of the Transformer model.

②Methods using the Transformer model between the encoder and decoder.

③Methods utilizing the decoder based on the Transformer model-based decoder methods.

The first class of methods aims at extracting higher-quality coded features by taking advantage of the Transformer model's strength in modeling remote dependencies. Therefore, some studies have directly adopted a Transformer as an encoder. TransUNet is a successful attempt to introduce a Transformer into medical image segmentation tasks [29]. This model employs a Transformer as an encoder, which combines the strengths of a Transformer and U-Net and can extract the global context from the labeled image chunks. At the same time, the Transformer helps to capture spatial relations over long-range distances. Compared with V-Net, AttnUNet, and ViT, TransUNet performs better on multi-organ and heart segmentation tasks. In this way, TransUNet can handle large image sizes without the memory constraints of traditional encoder–decoder models. Similarly, there are many models inspired by the U-shaped architecture described above. For example, Atek [30] et al. designed a two-scale encoder (Swin-Transformer) U-shaped architecture (SwinT-Unet) to integrate the Shift Window (Swin) Transformer module and the Transformer Interactive Fusion (TIF) module. Models incorporating hierarchical SwinT modules into the decoder include UNETR [31], Swin-Unet [32], TransClaw U-Net [33], MISSFormer [34], and others. In order to aggregate features from multiple scales of an image, many methods propose a Transformer model block based on parallel shift windows to improve SwinT. For, Feng et al. [35] proposed the ConvWin-UNet structure, which combines the ConvWin, Transformer, and UNet and utilizes the W-MSA (weighted multi-scale aggregation) mechanism and convolution operation to accelerate the convergence and enrich the information exchange between patches. Using convolutional window operations for each convolutional layer in the encoder and decoder, the model achieves an Average DSC of 79.39% and an HD of 21.39 mm in the Synapse dataset. And to deal with multiple related tasks simultaneously, some methods U-Net based on the introduction of numerous parallel branches, such as Wang [36] designed a hybrid MT-UNet network; MTM first computes the self-factor efficiently by Local–Global Gaussian Weighted Self-Attention (LGG-SA) and then mines the interconnections between data samples by external attention (EA). The MT-UNet model achieved 78.59% and 90.43% DSC on the Synapse and ACDC datasets, respectively. Finally, a U-shaped model is constructed for accurate medical image segmentation. The method consistently outperforms Trans-Unet and other visual Transformers for complex-shaped organ segmentation (e.g., liver and left kidney).

Unlike the above approaches, the second class of techniques aims to enhance the network's modeling capability in cross-layer feature transfer by incorporating a Transformer between the encoder and decoder to improve the performance of the segmentation task. For example, Zhou et al. [37] proposed a new 3D Transformer model called nnFormer. nnFormer introduces a self-attention mechanism based on local and global volumes to learn 3D volume representations and uses skip attention instead of skip connections to improve further the performance, which can be used to use less computational cost to model global feature relationships efficiently. The model achieved 86.4%, 86.57%, and 92.06% DSC on the BraTs2016, 2017, Synapse, and ACDC datasets. Outperformed the LeViT-UNet-384s and TransUNet and was more advantageous in segmenting the pancreas and the stomach in terms of mean HD and DSC, respectively. Similarly, introducing the cross-attention cross-convolution Transformer module instead of skip connections are DAE-Former [38], DSTUNet [39], and so on.

The third class of methods incorporates the Transformer into the encoder. For example, the Trans-U model proposed by Guo et al. [40] uses the combined high-resolution positional data from CNN features and the global context stored by the Transformer to compensate for the loss of feature resolution caused by the Transformer. The DSC result of this model on the Synapse dataset is 76.56%, which is lower than the U-Net and attnUNet models. The main reason is that the Transformer cannot extract low-level morphological details in medical images. However, it works well in capturing high-level semantic information that helps classify. For this reason, it is proposed to combine the Transformer with U-Net and let the Transformer learn the positional features through the jump connection of U-Net so that the model can utilize the high-level semantics as well as consider the low-level morphology and may obtain better results in medical image segmentation.

Unlike the U-shaped model-based approach mentioned above, to enhance the Transformer network's ability in local feature extraction, Wang et al. [41] proposed the use of a pyramid structure to construct multiscale representations and deal with multiscale variations, firstly, using a lightweight convolutional layer to extract the low-level features and reduce the amount of data, and then, using the Transformer block and the convolution block's mixture of Transformer blocks and Convolutional blocks to handle high-level features. Models with similar ideas include ECT-NAS [42], C2Former [43], CASTformer [44], etc. Niu et al. [45] proposed a novel symmetric supervised network based on the traditional two-branch approach, which utilizes a symmetric supervisory mechanism to enhance the supervision of the network training and introduces a Transformer-based global feature alignment module to improve the global consistency between the two branches. Compared with the baseline SE-Net [46], the method improved by 16.9% and 25.98% on the MS-CMRSeg and CHAOS datasets, respectively, and showed significant performance in the multi-organ left and suitable kidney segmentation experiments with 78.46% and 81.45%, respectively. To solve the problem of information loss or resolution degradation due to downsampling or cutting of the input image by traditional Transformer, Themyr et al. [47] proposed a full-resolution memory (FINE) Transformer model, which learns the memory Token by learning the memory Token, which scales well in terms of memory and computational cost, and allows for localized image segmentation. It scales well and interacts with local image regions and all 3D volumetric regions. FINE has better performance and superiority over CNN and recent Transformer model baselines (e.g., CoTr [48] and nnFormer [37]) to focus entirely on high-resolution images. FINE obtained 87.1% DSC and better segmentation of small and complex organs such as the pancreas (Pa) and gallbladder (Gb).

Furthermore, to reduce the dependence on expensive labeled kidney data and to be more efficient in data acquisition, Wang et al. [49] proposed a cross-teaching semi-supervised medical image segmentation model based on CNN and Transformer models, aiming to improve the efficiency of automatic segmentation of multiple organs in abdominal CT. However, it was found in the validation on the FLARE2022 challenge dataset that the segmentation effect could have been more satisfactory. Although the separation network could segment most organs, the location of organs such as kidneys shifted. In contrast, Xin et al. [50] used U-Net, the backbone network of nnU-Net [51], as the final prediction network based on the combination of CNN and Transformer. An average DSC of 75.80% was obtained in the FLARE2022 challenge. To perform accurate organ segmentation without the need for manual annotation, Wang et al. [52] designed a self-supervised learning-based framework for one-time kidney organ segmentation, which is used to build a network model of global correlation between the reference samples (VALUE) and the desired segmentation samples (QUERY). Local features are extracted using a CNN, and then global features are removed from the local feature space via a Transformer. A semantic dependency embedding method introduces channel and spatial standard information into the Transformer to establish global corrections. The experiment compares the model with PANet [53], SENet [54] and SSL-ALPNet [55], and the test scenarios include observed organ settings (OO) and unobserved organ settings (UO). The results show that the model outperforms the others in the MICCAI 2015 CT and ISBI2019 MRI datasets. This demonstrates the effectiveness of using self-supervised learning to train Transformer and Convolutional Hybrid Networks to handle better OO and UO scenarios in medical image segmentation tasks.

#### Segmentation of renal tumors

Renal tumor segmentation refers to accurately segmenting the tumor region in the kidney from the surrounding normal tissue in medical images to quantitatively identify and locate the location and extent of the renal tumor, which can effectively assist doctors in tumor diagnosis, treatment, and monitoring.

To further improve the segmentation and save the running time and memory of the algorithm. Some approaches apply a Transformer to the encoder for feature extraction of kidney images. For example, Yu et al. [56] proposed UNesT, which employs a simplified and faster converging Transformer model encoder design to achieve local communication between positional information by hierarchically aggregating spatially adjacent patch sequences. The model performs state-of-the-art on the four datasets BTCV, KiTS2015, BraTS2021, and KiTS2021, outperforming the state-of-the-art integrated model SLANT [57] in a whole-brain segmentation task. Some methods learn more straightforward mappings, focusing on normalized pose and size images. For example, Barbera et al. [18] proposed a new CNN architecture that contains three consecutive modules: a regression module, a differentiable module, and a segmentation module. The architecture uses a spatial Transformer model network (STN) to normalize the input image to improve the accuracy of subsequent segmentation tasks. The differentiable module automatically localizes the regions of interest to reduce the manual labeling effort. The segmentation module uses a UNet-based architecture, and the model achieved good DSC scores (88.01% for kidneys and 87.12% for tumors) in the segmentation task for kidneys and tumors on pediatric data and KiTS19 data. Inspired by the hierarchical structure in the visual Transformer model, Yu et al. [58] proposed a method to segment kidney components using a 3D block aggregation Transformer model. They constructed a kidney substructure segmentation dataset containing 116 subjects. The model enables localized communication between sequential representations without changing the self-attention mechanism. It showed advanced performance in the segmentation task with a DSC metric of 84.67%. Boussaid et al. [59] used the spatial Transformer model and linear subspace projection to compare segmentation masks in feature space and to characterize global shape properties. The authors experimented on a 3D ultrasound dataset of left and right adult kidneys from 667 patients and obtained a DSC metric of 92.07%, demonstrating the validity and accuracy of the method.

Chen et al. [17] proposed a multi-stage 2.5D semantic segmentation network for multi-stage fine segmentation to address the high cost of computational resources for kidney mass segmentation. The first stage uses ResSENormUnet [60] combined with deep residual connectivity and attention mechanism to pre-segment the kidney and predict the approximate location and shape. In the second stage, fine segmentation is performed using the DenseTransUnet [61] network combined with dense connectivity and self-attention mechanism to more finely segment the contours of the kidney, tumor and cyst. Finally, post-processing operations based on 3D-connected regions remove possible false-positive segmentation results. The model obtained good DSC for kidney segmentation (Kidney: 94.3%, Tumor:77.79%, Cyst:70.99%), but the network approach can be improved for segmenting smaller kidneys, tumors, and cysts. To enhance the spatial modeling capability of the network while maintaining the efficient use of computational resources, Yang et al. [62] proposed that the EPT-Net network effectively combines the edge sensing and Transformer structures and introduces the Dual Position Transformer to enhance 3D spatial localization capability. Meanwhile, the Edge Weight Guidance module extracts edge information without additional network parameters. Good performance is demonstrated on the relabeled KiTS2019 dataset (KiTS19-M).

#### Outlining of the renal target area

Radiation therapy is one of the most crucial localized treatment modalities for abdominal malignancies (e.g., cervical, prostate, pancreatic, renal, and liver cancers). Depicting abdominal organs at risk (OARs) on CT images is essential during radiation therapy management [63]. The method currently used in clinical practice is manual contouring of CT images, which is often very tedious and time-consuming. The results also vary depending on the skill level of the observer, environment, or equipment type. Deep learning-based automated contouring techniques for segmenting OAR would help eliminate these problems and produce consistent results with minimal time and labor [64].

Traditionally, there are conditional generative adversarial network (GAN) techniques proposed by Seenia et al. [64] for semantic segmentation of OAR in CT images of organs such as kidneys and Pan et al. [65] for multi-organ segmentation of abdominal CT images utilizing a V-net-like structure, a U-shaped multilayer perceptron mixer (MLP-Mixer) and a convolutional neural network (CNN). These methods need to use the image feature information effectively. At the same time, Jiang et al. [66] proposed the MRRN-NBSA method incorporating self-attention to segment multiple key OARs of head and neck (HN) and abdominal organ (BTCV) datasets. Comparison of MRRN-NBSA with Unet using cross-attention (CCA), dual-SA, and transformer-based (UNETR) methods showed that MRRN-NBSA obtained a DSC of HN: 88% and BTCV: 86%. The technique applies NBSA in a decoder that incorporates interactions between regional contexts while extracting non-local attentional information in a fast and memory-efficient manner. Overall, the network extracts relevant feature sets to generate accurate segmentation of organs such as kidneys by combining a deep multiresolution residual network and nested block (SA) self-attention to take advantage of multiscale features and self-attention mechanisms. To address the limitations in global and local information feature fusion in the classical TransUnet model decoder, Jiang et al. [67] proposed BiFTransNet, which introduces the BiFusion module into the decoder stage to achieve effective global and local feature fusion by enabling feature integration from various modules. It is used in the Synapse dataset to develop automated gastrointestinal image segmentation to help radiation oncologists accurately target the X-ray beam to the tumor.

#### Summary of segmentation algorithms

A literature search reveals that TransUNet, Swin-Unet, AgDenseU-Net 2.5D, LeViT-UNet, ViTBI, UNETR, and HiFormer are the more popular algorithms in the field of renal medical image segmentation at present and show different degrees of advantages in kidney image segmentation tasks. We conducted a comprehensive evaluation of the above segmentation algorithms, as shown in Table 1.

**Table 1:** Comparison of kidney image segmentation algorithm performance

Algorithms	Datasets	Evaluation indicators/results	Main views and contributions	Limitations
TransUNet [29]	Synapse 2015/ACDC	Synapse (DSC: 77.48%; Kidney (R): 81.87%; Kidney (L):77.02%; HD: 31.69 mm)/ACDC(DSC: 89.71%)	TransUNet is the first successful attempt to introduce a Transformer into medical image segmentation. Combining CNN and Transformer in coding	Transformer leads to a dramatic increase in the number of model parameters
IB-TransUNet [68]	Synapse 2015	DSC: Kidney (R):79.87% Kidney (L):83.89%	Using the UNet model to combine the information bottleneck (IB) with the Transformer	More advantages in learning small organ features
Swin-Unet [32]	Synapse 2015	DSC: 79.13% HD: 21.55 mm	The information bottleneck block was innovatively introduced in the encoding; a hierarchical Swin Transformer model with moving windows is used as an encoder to extract contextual features. An asymmetric Swin Transformer model decoder with a patch extension layer is designed to perform the upsampling operation	Higher dependency on large and diverse datasets with a large number of parameters and complexity
AgDenseU-Net 2.5D [60]	KiTS 2021	DSC: Kidney: 95% Tumor: 87.8% Cyst: 74.6%	Combining the features of AggRes (which enhances feature representation by aggregating residual connectivity and attention mechanisms) and DenseU-Net (which efficiently performs multi-scale feature fusion)	Higher computation and memory consumption, longer training time
LeViT-UNet [69]	Synapse/ACDC	Synapse (DSC: 78.53%, Kidney (R): 80.25%, Kidney (L): 84.61%, HD: 16.84 mm)/ACDC (DSC: 90.32%)	Using LeViT as the encoder of LeViT-UNet, combining LeViT Transformer with U-Net	Some metrics do not reach SOTA, and the segmentation performance is imaged to some extent to reduce the computational complexity
ViTBIS [70]	Synapse 2015	DSC: 80.45%	Adding the Concat operator for merging features	The dataset is more homogeneous, with fewer baselines for comparison
TransClaw U-Net [33]	Synapse 2015	Synapse (DSC: 78.09%, HD: 26.38 mm)	Claw U-Net with Transformer Combined/decoder dual-path design	Relatively homogenous data sets
After-Unet [71]	Thorax-85/BCV/SegTHOR thorax	Thorax-85 (DSC: 92.32%)/BCV (DSC: 81.02%)/SegTHOR thorax (DSC: 92.10%)	Both intra- and inter-slice long-distance cues were considered to guide segmentation	Axis information is naturally provided mainly for 3D volume
TransBTSV2 [19]	KiTS 2019/ BraTS2019/ BraTS2020/ LiTS 2017	KiTS 2019 (DSC: KIdney: 97.37%, Tumor: 83.69%, Composite: 90.53%)	Not limited to brain tumor segmentation (BTS) but focuses on general medical image segmentation, providing a powerful and efficient 3D baseline for the volumetric segmentation of medical images	Mainly for 3D medical image segmentation tasks
UNETR [31]	BTCV/MSD	BTCV (AVG: 89.1%)/MSD (DSC: 71.1%, HD95: 8.822 mm)	The Transformers encoder utilizes embedded 3D corpora to capture remote dependencies efficiently; the jump-join decoder combines extracted representations of different resolutions and predicts the segmentation output	Mainly for 3D medical image segmentation
DBT-UNETR [72]	BTCV	AVG:80.3%	An improved SwinUNETR is proposed based on UNETR with Swin Transformer as an alternative to Transformer	No significant improvement in performance compared to UNETR
NnFormer [37]	Synapse 2015/ ACDC	Synapse (DSC: 87.40%)/ACDC(DSC: 91.78%)	Utilizing a combination of cross-convolution and self-attention operations	Little performance gain on the ACDC dataset
HiFormer [73]	Synapse 2015	DSC:80.69%	Two multi-scale representations were designed based on the Swin transformer module and CNN encoder, and the Double-Level Fusion (DLF) module was designed to finely fuse the global and local features of the two representations	Single dataset
MPSHT [74]	Synapse 2015/ ACDC	Synapse (DSC: 79.76%, KIdney: 80.77%, HD: 21.55 mm)/ACDC(DSC: 91.80%)	Based on the CNN-Transformer model hybrid model, to which the asymptotic sampling module is added	Accuracy of segmentation to be improved
DSGA-Net [75]	Synapse 2015/ BraTs 2020/ ACDC	Synapse (DSC: 81.24%)/BraTs2020 (DSC: 85.82%)/ACDC(DSC: 91.34%)	Add a Depth Separable Gating Visual Transformation (DSG-ViT) module to the code and propose a Hybrid Three-Branch Attention (MTA) module	Considerable computational burden; consumes large amounts of GPU memory
MedNeXt [76]	BTCV/AMOS22/KiTS19/BraTS21/AVG	BTCV (DSC: 88.76%)/AMOS22 (DSC: 91.77%)/KiTS19 (DSC: 91.02%)/BraTS21 (DSC: 91.49%)/AVG (DSC: 88.01%)	The use of ConvNeXt 3D and the extension of ConvNeXt blocks to upsampling and downsampling layers represents a modern deep architecture for medical image segmentation	Deep Networks Dedicated to Medical Image Segmentation
MESTrans [77]	COVID-DS36/GlaS/Synapse/I2CVB	COVID-DS36 (DSC: 81.23%)/GlaS (DSC: 89.95%, IoU: 82.39)/Synapse (DSC: 77.48%, HD:31.69 mm)/I2CVB (DSC: 92.3%, IoU: 85.8)	Propose a Multi-scale Embedding (MEB) and Multi-layer Spatial Attention Transformer structure (SATrans) to adjust the sensory field. Propose a Feature Fusion Module (FFM) for global learning between shallow and deep features	The performance of small organ segmentation needs to be improved
ST-Unet [78]	Synapse 2015/ISIC 2018	Synapse2015(DSC:78.86%, HD:20.37mm)/ISIC 2018(F1:90.94%, mIoU:85.26)	Proposing a new Cross-Layer Feature Enhancement (CLFE) module for cross-layer feature learning with spatial and channel squeezing and excitation modules to highlight the saliency of specific regions	The accuracy of segmentation needs to be improved
COTRNet [79]	KiTS 2021	DSC: Kidney:92.28% Tumor:55.28% Cyst:0.50.52%	Utilizing pre-trained ResNet to develop the encoder, in addition to adding deep supervised	The accuracy of segmentation for masses and tumors needs to be improved
CS-Unet [80]	Synapse 2015	DSC:82.21% Kidney(R):79.52% Kidney(L):85.28%	Design of convolutional Swin-Transformer (CST) module that merges convolution with multi-head self-attention and feed-forward networks	Facing the challenge of dealing with long-range dependencies

### Transformer applied to kidney image classification

Kidney image classification is categorizing kidney image data into different categories or labels. With deep learning technology, kidney images can be automatically analyzed and classified to provide more accurate and faster diagnostic results. This helps to improve the early detection and treatment of kidney diseases. Due to the complexity of morphological and structural features of kidneys and surrounding tissues, the task of renal image classification usually needs to consider different levels of features [81], including renal morphology, size, texture, and so on. Traditional CNN models have limitations in dealing with complex kidney morphological and structural features. In contrast, Transformer can extract multiple sets of feature representations in parallel and incorporate a fully connected layer to fuse and classify the features, thus improving the model performance [82]. Therefore, applying Transformer to the renal image classification task can improve the accuracy and sensitivity, especially for the classification of renal cysts, tumors, stones, etc., thus helping doctors to understand renal lesions more accurately and provide better treatment plans and prognosis assessment.

#### Classification network model based on the combination of Transformer and CNN networks

The first class of approaches applies the Transformer to an encoder–decoder structure, where the encoder–decoder consists of multiple identical layers, each containing an Attention mechanism and a feed-forward neural network. For example, the MT-ONet network [83], combines CNN, hybrid Transformer and LGG-SA into the encoder component of the proposed O-Net architecture to improve the classification accuracy. The second class of approaches uses the Attention mechanism between the encoder and decoder to capture the dependency between input and output. For example, the CTransPath [84] network uses a new Semi-Supervised Learning (SSL) strategy called Semantic Relatedness Contrastive Learning (SRCL), which utilizes the local features of CNNs mining capability and the global interaction capability of Transformer, which has some advantages in solving small sample data.

In diagnostic pathology, whole-slice images are typically huge and often have only overall labels and no labels corresponding to specific instances (e.g., cells or lesions). This leads to the fact that traditional supervised learning methods cannot be directly applied to this problem. To transform the weakly supervised classification problem into an overlooked learning problem, Shao et al. [16]. proposed a new framework called Multiple Instance Learning (MIL) to explore the correlation between different instances to solve the weakly supervised classification problem in pathological diagnosis based on the whole section images of the kidney, based on the MIL framework, the paper designs A Transformer model-based MIL (i.e., TransMIL), which can efficiently handle unbalanced/balanced and binary/multiple classification with good visualization and interpretability. TransMIL achieved an AUC of 93.09% and TCGA-NSCLC: 96.03% TCGA-RCC: 98.82% on the CAMELYON16 and TCGA datasets.

CNNs are more commonly used for renal image classification tasks than Transformer models; for example, Cicalese et al. [85] proposed an uncertainty-guided Bayesian Classification (UGBC) scheme for glomerular and renal level classification tasks. Qadir et al. [86] used a deep migration learning model based on the DenseNet201 network to classify the tumor, normal cysts and stone regions of the kidney. Aruna et al. [87] used networks such as CNN and VGG19 to diagnose polycystic kidneys, and the classification task covered cysts, tumors, and stones. Hossain et al. [88] used three classification methods, namely, EAnet, ResNet50, and a customized CNN model, to classify the four types in CT images of the kidney (cysts, normal, stones, tumors). Chanchal et al. [89] proposed the RCCGNet network for fully automated renal cell carcinoma grading from renal histopathology images.

#### Summary of classification algorithms

In kidney image classification, algorithms based on CNN or combining CNN and Transformer have become a hot research topic. These algorithms utilize the feature extraction capability of CNN and the sequence modeling capability of Transformer to improve the accuracy and efficiency of kidney image classification. In this paper, we summarize some crucial algorithms, including TransMIL, CTransPath and other algorithms and CNN and DNN-based algorithm models, and their performance is summarized and compared in detail in Table 2. This provides an opportunity to analyze their strengths and limitations in depth and provides a reference for future research and applications.

**Table 2:** Comparison of kidney image classification algorithm performance

Algorithms	Datasets	Evaluation indicators/results	Main views and contributions	Limitations
TransMIL [16]	CAMELYON16/TCGA-NSCLC/TCGA-RCC	AUC: (CAMELYON16: 93.09%, TCGANSCLC: 96.03%, TCGA-RCC: 98.82%)	Using multiple instance learning (MIL) to explore morphological and spatial information in images	Mainly dealing with weakly supervised classification in whole-slice image (WSI)-based pathology diagnosis
CTransPath [84]	TCGA-RCC	AUC:99.1%	Self-computation of localized window attention using Swin-Transformer as a backbone model	Large amounts of unlabeled data are required
UGBC [85]	private dataset	ACC (glomerulus: 96.30%, Kidney: 96.60%)	Assigning image labels based on kidney-level classification using a high-throughput batch labeling scheme to exploit label noise immunity associated with deep neural networks (DNNs)	Dependence on the accuracy of label annotations
DenseNet201–Random Forest [86]	CT KIDNEY DATASET: Normal-Cyst-Tumor and Stone	ACC: 99.44% (cyst: 99.60%, kidney: 98.90%, tumor: 100%)	Feature extraction using deep migration learning model DenseNet-201-Random Forest	More resources are needed to train and use both models simultaneously
RCCGNet [89]	KMC-kidney dataset/BreakHis dataset	KMC-kidney (ACC: 90.14%, F1:89.06%)/BreakHis (ACC: 90.09%, F1: 88.90%)	RCCGNet contains a shared channel residual (SCR) block, which shares information between two different layers and complements each other's shared data	The model integration is complex

### Multi-modal image alignment

Multimodal image alignment is aligning and matching renal image data from different modalities. By aligning images from other modalities, the correlations and implied relationships between them can be revealed, providing researchers with more information and insight. In clinical practice, doctors often need to refer to renal image data from multiple modalities simultaneously, such as MRI, CT, and ultrasound images. By aligning these images, the correlation analysis between different modalities can be realized, improving the accuracy of diagnosis and treatment decisions.

Chi et al. [90] proposed a new depth alignment pipeline for free-breathing 3D CT and 2D ultrasound (U/S) kidney scans. The pipeline consists of a feature network and a 3D–2D CNN-based alignment network. The feature network has hand-textured feature layers to reduce semantic gaps. The alignment network adopts the encoder–decoder structure of feature image mismatch (FIM), is first pre-trained with a retrospective dataset and training data generation strategy, i.e., the kidneys are uniformly aligned on the upper and lower axes on the CT images, and then the kidneys are aligned with the center of mass on the U/S images, and successfully achieves accurate alignment between kidneys on CT and U/S images. The pipeline solves the challenge of 3DCT–2DUS kidney alignment during free-breathing with a new network structure and training strategy and obtains a DSC of 96.88% and 96.39% in CT and U/S images, respectively.

## Other clinical applications for transformer

In addition to intelligent analysis and intelligent diagnosis of medical images, the Transformer mechanism can also be applied to renal image detection, disease prediction, image alignment, electronic reports related to renal diseases, clinical decision models, etc. [91]. These renal image processing tasks involve large and complex image data, and the models constructed by traditional convolutional neural networks can hardly meet the actual clinical needs. Using an improved Transformer for kidney image data application is an efficient strategy that can help the medical imaging field accomplish quantitative analysis and clinical diagnosis of kidney images more accurately [92].

### Transformer application for kidney disease prediction

The main clinical applications of renal ultrasonography include ruling out reversible causes of acute kidney injury, such as urinary tract obstruction, or identifying irreversible CKD to rule out unnecessary tests, such as renal biopsy [93]. Traditional methods of assessing kidney injury have relied on metrics such as kidney length, volume, cortical thickness, and echogenicity [94]. However, in recent years, advances in deep learning and computer vision have enabled machine learning and artificial intelligence techniques to more accurately and objectively assess kidney images, providing more comprehensive information to diagnose kidney injury and treatment decisions. Compared to traditional qualitative or semi-quantitative assessment methods, these techniques can reduce the influence of operator experience and subjective factors and provide more accurate assessment results [95].

Ma et al. [96] used a novel multimodal data model combining Transformer's bi-directional encoder representation and optical gradient boosters to improve CKD prediction. The MD-BERT-LGBM model was used in a CKD prediction experiment using over 3 /ls of medical data from 3295 participants and compared with traditional LR, LGBM and multimodal disease risk prediction algorithms. The results showed that MD-BERT-LGBM is expected to play an essential role in predicting and preventing CKD for clinical applications. Zeng et al. [97] constructed a sequential model for the prediction of acute kidney injury (AKI) induced by sepsis in the ICU. The attention-based sequential conduction model outperforms logistic regression, XGBoost, and RNN through a comprehensive performance evaluation. Its AUROC is 79.5% and AUPRC is 65.0%. Asif et al. [7] proposed a deep migration learning architecture based on the pre-trained VGG19 [98] model and Inception module, i.e., the architecture of the VGG19 model was customized by removing the fully connected layer and placing a randomly initialized plain Inception module and other coatings. It is used to detect major renal diseases from CT images. The experiments considered two migration learning approaches: feature extractor and fine-tuning. An AUC of 99.25% was achieved on 4000 renal CT images. The proposed model is of great benefit to urologists in detecting renal diseases. Shickelae et al. [99] designed a multi-stage end-stage renal disease (ESRD) prediction framework for ESRD based on the Transformer model. The framework was based on nonlinear dimensionality reduction, relative Euclidean pixel distance embedding, and spatial self-attention mechanisms for predictive modeling. Researchers developed a deep transformer network for coding WSI and predicting future ESRD using a dataset of 56 renal biopsy WSIs from patients with diabetic neuropathy at Seoul National University Hospital. The subjects had an AUC of 97% for the prediction of 2-year ESRD. Aboutalebi et al. [21] designed a clinician assessment-based dataset containing clinical and biochemical data of 1366 patients. Different machine learning models were developed and trained to predict kidney injury, including gradient-based augmented tree and deep Transformer architecture.

### Transformer in electronic reporting

Electronic reporting has also been progressively applied in the medical field. Schuppe et al. [23] used the large-scale language Transformer model open source artificial intelligence ChatGPT, a patient diagnosed with bilateral renal cell carcinoma who underwent right partial and left total nephrectomy as well as episodic biliary atresia (BA) exhibited nephrotic syndrome (NS) signs and symptoms article reports were written. Yang et al. [24] described a methodology to develop a language model for reporting renal transplant pathology. The study aimed to answer two predefined questions: what rejection did the patient exhibit, and what was the grade of interstitial fibrosis and tubular atrophy (IFTA)? For this purpose, a corpus containing 3.4K renal transplant ports and 1.5 million words were used in the paper for pre-training in clinical BERT and fine-tuned with QA headers. Additionally, an extended renal BERT (i.e., exKidneyBERT) model was created, pre-trained and fine-tuned using the same corpus to capture the complex vocabulary of a narrow technical domain.

### Application of transformer in decision-making systems

Zhang et al. [22] utilized the Decision Transformer model, an offline RL (reinforcement learning) paradigm for continuous time decision-making in the healthcare domain. In the paper, the model was generalized to a continuous-time decision-making scenario, considered past clinical measurements and treatments, and learned methods for suggesting future visit times and per-treatment schedules. Experimental results show that the continuous-time decision-making Transformer model can outperform its competitors. It has clinical utility in improving patients' health and prolonging their survival by learning high-performance strategies from log data generated using strategies of different quality levels.

### Other applications summary

Kidney images play an essential role in clinical applications, and different algorithms have been proposed to achieve kidney image alignment and disease detection. Table 3 compares the performance and usage of several standard algorithms for clinical applications of kidney images.

**Table 3:** Performance comparison of kidney image algorithms for other applications

Algorithms	Datasets	Evaluation indicators/results	Main views and contributions	Usage
VGG19 [87]	Private dataset	ACC: 98%	VGG19 uses a deeper network structure and a small convolutional kernel for improved feature extraction	Kidney cysts detection
EANet [88]	CT KIDNEY DATASET: Normal-Cyst-Tumor and Stone	ACC: 83.65%	Introduction of attention mechanism, multi-scale feature fusion, efficient network design, cross-layer feature interaction	Kidney cyst classification
ResNet50 [88]	CT KIDNEY DATASET: Normal-Cyst-Tumor and Stone	ACC: 87.92%	Having introduced Residual Block and Batch Normalization	Kidney cyst classification
MD-BERT-LGBM [96]	private dataset	ACC: 78.12% AUC: 85.15%	The model integrates a bi-directional encoder representation of the Transformer with an optical gradient lifter, a multimodal data model	CKD disease prediction
KidneyRegNet [90]	KiTS19/in-house datasets	KiTS19 (DSC: 96.88%, Sensitivity: 0.9711, Specificity: 0.9667)/in-house (DSC: 96.39%, Sensitivity: 0.9736, Specificity: 0.9560)	A new depth-alignment pipeline for free-breathing 3D CT and 2D U/S renal scans is proposed	Kidney alignment
ChatGPT [23]	NA	NA	The core algorithm is the Transformer, which combines the Transformer model's self-attention mechanism with the language model's generative power	Nelson syndrome (NS) pathology report writing
MulGT [100]	TCGA-KICA/TCGA-ESCA	KICA (Typing: AUC: 98.44%, ACC: 93.89%, F1: 93.89%, Staging: AUC: 80.22%, ACC: 74.98%, F1: 72.55%)/ESCA (Typing: AUC: 97.49%, ACC: 92.81%, F1: 92.74%, Staging: AUC: 71.48%, ACC: 66.63%, F1: 65.73%)	A domain knowledge-driven graph pooling module was designed to simulate diagnostic patterns for different analysis tasks	WSI task diagnostics
Transformer [22]	DIVAT (Database of Kidney Transplantation Medical Records)	NA	For use in medical fields where continuous-time decision-making is required	Medical decision system
Transformer [99]	Dataset of 56 renal biopsy WSIs in patients with DN	AUC: 97%	A multi-stage ESRD prediction framework based on the Transformer model	For encoding WSI (whole-slice images) and predicting future ESRDs
Transformer [20]	Private dataset	F1: 96.3%, AUC: 98.9%	Predicting Kidney Transplant Function Using the Critical Mask Tensor of the Transformer Dot Product Attention Mechanism	Predicting kidney transplant function
COVID-Net [21]	Private dataset	Survival prediction: ACC:93.55%, Kidney Injury Complications: ACC:88.05%	Proposing an interpretability-driven framework for building machine learning models to predict survival and kidney injury in patients with no coronary pneumonia from clinical and biochemical data	Predicting survival and kidney injury in patients with new crown pneumonia
ExKidneyBERT [24]	Private dataset	OneQA (ACC: 83.3%) TwoQA (ACC: 95.8%)	Linguistic modeling of renal transplantation pathology reports	Renal pathology reports

## Discussion and outlook

This paper presents a comprehensive overview of Transformer model-based methods used for renal image processing tasks. After extensive comparisons and systematic analysis, compared with traditional CNNs, the Transformer model-based approach can capture the correlation between different locations in an image through the self-attention mechanism. It can consider global and local contextual information, improving the model's ability to understand and judge images. It shows excellent performance and potential to become the backbone network model in the renal disease image processing task.

In the clinic, the Transformer model-based approach can provide quantitative image analysis for doctors, thus assisting in the diagnosis and treatment planning of kidney disease. It has certain advantages in the segmentation and classification of kidney images: ① compared with other traditional models, the Transformer can effectively deal with long-range dependencies through the self-attention mechanism and can better capture the relationship between each part of the image, thus improving the accuracy of segmentation and classification; ② transformer model is more suitable for dealing with long sequence data and global information. The self-attention mechanism in Transformer allows interaction between arbitrary positional information without limiting parameter sharing and local sense fields, thus providing greater flexibility; ③ transformer model can be easily extended to handle multimodal data, such as the combination of image and text, which is advantageous in the task of multimodal information.

Although the Transformer model has unique advantages and potential in kidney image segmentation and classification tasks, some challenges and limitations must be addressed. For example, ① the Transformer may suffer from information loss when dealing with long-range dependencies compared to traditional CNNs;② the Transformer model consumes a large amount of computational resources, including memory and computational power, when dealing with large-scale image data. This may limit its feasibility and efficiency in practical clinical applications. ③ Transformer models usually require a large amount of training data for good generalization ability. However, in medical images, especially kidney images, acquiring large-scale labeled data is a challenging task.

Future research directions include the more effective integration of CNN and Transformer, the design of novel Transformer model architectures, the handling of multi-modal data, addressing unstructured data, and leveraging weak supervision and self-supervised learning to enhance the performance of clinical applications. The development of versatile and robust Transformer methods will facilitate improved analysis and application of clinical data. In the context of implementing this model in real-world medical diagnostics, three key challenges and considerations emerge. Firstly, privacy and security of data must be taken into account. Patient's private data should be appropriately handled and protected to prevent data leakage. Secondly, there is a need for diversity in training data. Currently, clinical sample sizes remain limited, resulting in constrained model generalization to different populations and disease types. Collaboration with more healthcare organizations is essential to collect large-scale clinical samples for model training to enhance its quality. It is worth noting that Transformer models typically require substantial training data to achieve strong generalization. However, obtaining extensive annotated data, especially in the field of medical imaging, such as kidney images, poses a challenging task. Lastly, it is necessary to validate the model's stability across multiple datasets, collecting diverse samples from different healthcare organizations for validation to test the model's robustness in various settings.

Through our review, we recognize the crucial importance of preprocessing methods in current kidney CT image processing. In our future work, we plan to further optimize and propose more effective CT image preprocessing approaches to overcome current challenges and limitations. Our method involves multi-step data preprocessing, including voxel size resampling, grayscale normalization, noise reduction, contrast enhancement, histogram equalization, region cropping, and data augmentation techniques. These comprehensive preprocessing steps aim to optimize model input, enhance performance, and improve generalization capabilities.

## Conclusion

In kidney image analysis, diverse architectures and optimization techniques have significantly improved model performance. Transformer architectures for kidney image analysis are typically optimized in three main aspects: ① hybrid CNN and Transformer models, such as TransUnet [29]and U-Net variants, are employed to extract local features and learn global dependencies; ② introduction of 3D Transformer architecture, e.g., TransBTSV2 [19], focuses on learning CT/MRI 3D structural relationships, proving advantageous in volumetric image analysis compared to 2D models; ③transformer model modifications, including attention mechanism updates and depth increase for richer feature learning. For instance, the DSGA-Net [74] model introduces a Depth Separable Gated Visual Transformer (DSG-ViT) module to learn deeper features of kidney images. Multimodal data fusion, exemplified by MD-BERT-LGBM, combines different imaging modalities (CT, MRI, ultrasound) and text/label data, enhancing feature characterization. In summary, to enhance kidney image analysis task performance, appropriate model architectures need to be selected or modified based on data and task characteristics. We have summarized the features and performance of each model, providing a valuable reference resource for advancing and expanding kidney image analysis research.

## Data Availability

Not applicable.

## Abbreviations

CNN: Convolutional neural network
CKD: Chronic kidney disease
KSD: Kidney stones disease
CT: Computed tomography
MRI: Magnetic resonance imaging
RNNs: Recurrent neural networks
ViT: Vision Transformer
TIF: Transformer interactive fusion
W-MSA: Weighted multi-scale aggregation
LGG-SA: Local–global Gaussian-weighted self-attention
EA: External attention
FINE: Full-resolution memory
STN: Spatial Transformer model network
FCN: Full convolutional network
OARs: Organs at risk
GAN: Generative adversarial network
MLP-Mixer: U-shaped multilayer perceptron mixer
HN: Head and neck datasets
BTCV: Abdominal organ datasets
KiTS 2019: Kidney Tumor Segmentation Challenge 2019
KiTS 2021: Kidney Tumor Segmentation Challenge 2021
Synapse 2015: Synapse Multimodal MRI Segmentation and Classification Challenge 2015
ACDC: Automated Cardiac Diagnosis Challenge
Thorax-85: Thoracic Disease Screening in Chest Radiographs Dataset and Challenge
BCV: Brain Cancer Vision
SegTHOR thorax: Segmentation of THoracic Organs at Risk
BraTS2019: Multimodal Brain Tumor Segmentation Challenge 2019
BraTS2020: Multimodal Brain Tumor Segmentation Challenge2020
LiTS 2017: Liver Tumor Segmentation Challenge 2017
MSD: Medical Segmentation Decathlon
AMOS22: AMOS Medical Image Analysis Challenge 2022
BraTS21: Multimodal Brain Tumor Segmentation Challenge 2021
COVID-DS36: COVID-19 Diagnosis using Chest X-ray Images Dataset and Challenge
GlaS: Glasgow Retinal Image Analysis Challenge: Image Registration
ISIC 2018: 2018 International Skin Imaging Collaboration: Skin Lesion Analysis Towards Melanoma Detection Challenge
DIVAT: Database of Kidney Transplantation Medical Records
CAMELYON16: Camelyon16: A Benchmark for Fully Automatic Multi-Path Segmentation of Lymph Nodes
TCGA: The Cancer Genome Atlas
SSL: Semi-supervised learning
SRCL: Semantic relatedness contrastive learning
MIL: Multiple instance learning
UGBC: Uncertainty-guided Bayesian Classification
U/S: Ultrasound
FIM: Feature image mismatch
AKI: Acute kidney injury
ESRD: End-stage renal disease
BA: Biliary atresia
NS: Nephrotic syndrome
IFTA: Interstitial fibrosis and tubular atrophy
MS-CMRSeg: MICCAI 2019 Multi-sequence Cardiac MRI Segmentation Challenge
CHAOS: ISBI 2019 Combined Healthy Abdominal Organ Segmentation Challenge
